# Epistemic injustice in a case of cyclic vomiting syndrome. A case report

**DOI:** 10.1192/j.eurpsy.2021.663

**Published:** 2021-08-13

**Authors:** A. Cerame Del Campo, P. Coucheiro Limeres, A. Franco Soler

**Affiliations:** Centro De Salud Mental, Instituto Psiquiátrico José Germain, Leganes, Spain

**Keywords:** cyclic vomiting syndrome, epistemic injustice, testimonial injustice, hermeneutical injustice

## Abstract

**Introduction:**

We present the case of a 19-year-old female patient treated in our hospital due to an outburst of persistent vomiting. The patient had a diagnosis of Cyclic Vomiting Syndrome (CVS), a year before the diagnosis the patient had been labeled as a somatizer and admitted into the department of psychiatry. Given her psychiatric record and the fact that CVS is a rare diagnosis we were consulted on arrival.

**Objectives:**

CVS is an infrequent disorder of unknown etiology which shares similarities with migraine headaches. It is characterized by episodes of vomiting followed by periods of remission without active symptomatology with no organic pathology to account for the symptoms. Epistemic injustice (EI) is defined by Miranda Fricker as “a damage done to someone in their capacity as a knower”. She defined two forms of EI: testimonial and hermeneutical injustice.

**Methods:**

A case report is presented alongside a review of the relevant literature regarding CVS and epistemic injustice.

**Results:**

On arrival at the emergency department she tried explaining her condition, but her testimony was disregarded on the basis of her psychiatric record. It was only after the on-call psychiatrist explained the condition when she received the appropriate abortive treatment, after which she was admitted to the internal medicine department where she was followed by the liaison psychiatrist.

**Conclusions:**

CVS is a disabling disease still unknown to most clinicians in spite of the increasing quality evidence about its identification and treatment. The case highlight how cases of newly identified disease can suffer from testimonial and hermeneutical injustice.

